# NAH-GNN: A graph-based framework for multi-behavior and high-hop interaction recommendation

**DOI:** 10.1371/journal.pone.0321419

**Published:** 2025-04-29

**Authors:** Guangzhu Tan

**Affiliations:** 1 School of Big Data and Artificial Intelligence, Chongqing Institute of Engineering, ChongQing, China; 2 School of Big Data and Software Engineering, Chongqing University, ChongQing, China; National University of Defense Technology, CHINA

## Abstract

With the growing demand for personalized marketing, recommender systems have become essential tools to help users quickly discover products or content that match their interests. However, traditional recommendation methods face significant limitations in handling complex user behaviors and sparse data, particularly in accurately capturing relationships among diverse interaction types and higher-order dependencies. To address these challenges, this paper proposes a novel recommendation model based on graph neural networks (MBH-GNN) to optimize personalized marketing strategies. MBH-GNN constructs a multi-behavior interaction graph and employs neighborhood-aware modeling to effectively integrate diverse user-item interaction types (e.g., browsing, favoriting, purchasing), dynamically assigning weights to these behaviors to generate semantically rich embeddings. Furthermore, the model incorporates a high-hop relational learning mechanism to capture long-range user-item dependencies, enhancing its ability to model contextual information. These features enable MBH-GNN to achieve higher recommendation accuracy and diversity in complex scenarios. Experimental results demonstrate that MBH-GNN significantly outperforms existing baseline methods, achieving HR@10 of 0.789 and NDCG@10 of 0.330 on the BeiBei dataset, and HR@10 of 0.773 and NDCG@10 of 0.319 on the Tmall dataset. The model exhibits exceptional robustness and adaptability, particularly in addressing data sparsity and cold-start scenarios. This study offers an efficient and scalable solution for personalized marketing, providing critical theoretical support and practical value for improving recommendation system performance and addressing complex user behavior modeling challenges.

## Introduction

In the era of information explosion, the number of products and content on internet platforms has grown exponentially, offering users unprecedented choices [1,2]. However, faced with massive information and diverse needs, users often find it challenging to quickly locate the most relevant products or services. This phenomenon of "information overload" significantly affects user experience and becomes a critical obstacle to improving platform efficiency. Particularly in e-commerce, video streaming, and social media domains, accurately predicting user preferences and improving decision-making efficiency through recommendation systems has become a core competitive factor for platforms [3–5]. As an essential information filtering tool, recommendation systems have been widely applied in various scenarios, including e-commerce (e.g., Amazon, Taobao), streaming platforms (e.g., Netflix, YouTube), and social media (e.g., Facebook, Weibo) [6–8]. By analyzing users’ historical behaviors, recommendation systems predict potential user interests and provide personalized recommendations, thereby enhancing user satisfaction and increasing the platform’s commercial value. For example, e-commerce platforms leverage recommendation systems to boost click-through rates and sales, while social networks utilize personalized recommendations to enhance user interaction and extend usage duration [9,10]. However, the performance of recommendation systems is often constrained by challenges such as data sparsity, complex user behavior patterns, and cold-start scenarios, posing higher demands on the design and optimization of models.

Traditional recommendation methods are primarily based on collaborative filtering (CF) techniques, which mine potential interest patterns by analyzing user-item interaction matrices [11,12]. These methods are simple and efficient but perform poorly in highly sparse data scenarios or when users and items lack interaction records. To address this issue, latent factor models, such as matrix factorization (MF), have been developed to embed users and items into a low-dimensional space, capturing their underlying relationships [13,14]. However, these methods usually assume static user preferences and fail to deeply model the dynamic and diverse nature of user behaviors. Additionally, with the increasing scale of data and complexity of behaviors, traditional methods face significant challenges in scalability for complex scenarios. In recent years, the rapid advancement of deep learning techniques has introduced new research directions for recommendation systems. Neural-based recommendation models, such as neural collaborative filtering (NCF) and sequential recommendation models, learn nonlinear relationships between users and items, significantly improving recommendation accuracy [15–17]. These methods capture the dynamic characteristics of user behaviors and employ attention mechanisms to assign different weights to various behaviors [18]. However, existing methods typically focus on modeling single types of behavior, overlooking the semantic relationships among multiple behaviors. Furthermore, they struggle to capture high-order relationships or cross-level dependencies between users and items, limiting their effectiveness in complex recommendation scenarios. Graph neural networks (GNNs) provide a novel solution for recommendation systems. GNNs model the graph structures of users and items, effectively capturing high-order neighborhood relationships and cross-behavior dependencies, thereby enhancing recommendation accuracy and diversity [19,20]. For instance, methods such as NGCF, PinSage, and LightGCN employ message-passing mechanisms to learn high-order interaction relationships between users and items, alleviating data sparsity issues to some extent [21–24]. However, these methods predominantly focus on single-behavior or local relationship modeling, failing to fully utilize the dynamic weighting of multi-behavior signals and lacking the capability to capture long-range dependencies between users and items.

To address these challenges, this paper proposes a novel recommendation model called MBH-GNN (Multi-Behavior and High-Hop Graph Neural Network), aiming to enhance recommendation performance in complex scenarios comprehensively. MBH-GNN integrates multi-behavior interaction modeling, neighborhood-aware mechanisms, and high-hop relationship learning to provide innovative solutions for modeling diverse user-item interactions. The model is designed to capture rich relationships between users and items while maintaining high efficiency in the face of challenges such as data sparsity and cold starts.

The main contributions of this study are as follows:

- We propose the MBH-GNN model, which innovatively combines multi-behavior interaction modeling, neighborhood-aware GNNs, and high-hop relationship learning to improve the capability of recommendation systems to model diverse user behaviors.
- We construct a systematic experimental framework to comprehensively evaluate the model’s performance in real-world scenarios, including comparisons with state-of-the-art baseline models and in-depth analyses through ablation studies.
- We explore the contributions of modular designs to recommendation tasks, validating the rationality of the model’s architecture and offering new perspectives for optimizing recommendation systems.

The structure of this paper is as follows: Section Related work reviews reviews the related work in recommendation systems and GNNs and analyzes the limitations of existing methods. Section Methods describes describes the design of the MBH-GNN model, including the implementation of multi-behavior interaction modeling, neighborhood-aware mechanisms, and high-hop relationship learning. Section Experiment outlines outlines the experimental settings, including datasets, baseline models, evaluation metrics, and experimental designs, and presents the results and analyses. Finally, Section Conclusion wraps wraps up the study and explores potential avenues for future research.

## Related work

### The application of traditional methods in enterprise risk prediction

Traditional recommendation algorithms mainly include Collaborative Filtering (CF) and Content-Based Recommendation (CBR). Collaborative Filtering predicts user preferences for items based on their historical interactions, generating recommendations by leveraging the similarity between users (user-based CF) or items (item-based CF) [25–27]. These methods have shown great effectiveness in scenarios with sufficient data, providing accurate and relevant recommendations [28]. However, when user-item interaction data is sparse or completely missing, the performance of collaborative filtering degrades significantly, particularly in cold-start scenarios. Additionally, traditional CF assumes static user preferences, neglecting the temporal sequences and dynamic changes in user behavior, which limits its ability to capture diverse and evolving user interests [29].

Content-Based Recommendation methods, on the other hand, focus on analyzing the attributes or content features of items to match user preferences and generate recommendations. These methods are particularly effective in cold-start situations where user-item interactions are limited [30–32]. Compared to CF, CBR emphasizes item-specific information such as text descriptions, images, and category labels, providing explicit representations of user interests [33,34]. However, these methods often fail to account for the dynamic nature of user behavior and the intricate relationships between behaviors, limiting their ability to provide diverse and adaptive recommendations. Moreover, in real-world applications, the quality and completeness of item feature descriptions directly affect the accuracy of recommendations. Poor or missing information can lead to reduced generalizability and performance [35]. In recent years, hybrid recommendation methods have emerged as a promising solution to address the limitations of traditional approaches. These methods combine the strengths of CF and CBR to improve recommendation performance. For example, implicit feedback matrix factorization techniques (e.g., BPR, NMF) represent user-item interactions as low-dimensional embeddings and optimize ranking loss or rating prediction to enhance performance [36]. Additionally, some hybrid approaches incorporate contextual information, such as users’ geographical locations or temporal contexts, to further improve personalization and relevance [37,38]. However, even hybrid methods face challenges in capturing complex user behavior patterns, particularly when modeling high-order relationships and multi-layer interactions. These limitations restrict their application potential in dynamic and complex recommendation scenarios.

While traditional recommendation algorithms have served as foundational techniques in various scenarios, the rapid growth of data volume and the complexity of user behavior patterns have made it increasingly difficult to rely solely on CF or CBR methods. This has motivated researchers to explore new technical directions to address challenges such as data sparsity, cold-start problems, and complex behavior modeling, providing theoretical insights and practical guidance for advancing modern recommendation systems.

### Graph neural network-based recommendation algorithms

With the extensive application of GNNs in deep learning, this technology has gradually become a pivotal direction in recommendation system research. The prominent feature of GNNs is their ability to effectively capture complex relationships in graph structures and update and optimize node representations by recursively aggregating neighborhood information [39–42]. Particularly in recommendation systems, the interaction relationships between users and items naturally form heterogeneous graph structures, and GNN technology provides a powerful tool for modeling these complex interactions. Compared to traditional methods, GNNs can efficiently model higher-order neighborhood information in user-item interaction graphs, resulting in richer and more expressive node embeddings.

GNN-based recommendation algorithms can be categorized into two main types: GNNs designed for single-relation modeling and recommendation models based on multi-relation heterogeneous graphs. The former, such as GraphSAGE and GAT, aggregate neighborhood information via sampling to generate context-sensitive embeddings for users and items, demonstrating strong performance in various recommendation scenarios [43–45]. However, these approaches often overlook semantic differences and latent correlations between different types of behaviors, limiting their adaptability to multi-behavior scenarios. Recently, researchers have started exploring recommendation systems based on heterogeneous graphs, such as NGCF and PinSage, which incorporate multi-relation and multi-behavior information into GNNs [46]. By leveraging dynamic weighting and relationship modeling, these methods have further optimized recommendation performance. Particularly in large-scale industrial datasets, these methods have outperformed traditional collaborative filtering and matrix factorization approaches. Additionally, cutting-edge studies have investigated leveraging multi-hop feature propagation to uncover deeper graph structure information, demonstrating robust performance in handling sparse interaction data [47,48]. Moreover, GNNs have achieved significant progress in multi-hop relationship modeling. By recursively aggregating higher-order neighborhood information, GNNs capture long-range dependencies between users and items, thereby improving recommendation accuracy. Advanced models such as Heterogeneous Graph Neural Networks (HGNN) and Knowledge Graph Convolutional Networks (KGCN) incorporate knowledge graphs or multimodal features to enrich contextual support for recommendation systems [49–51]. Some studies also utilize the dynamic feature modeling capabilities of GNNs to provide more efficient solutions for real-time recommendation scenarios, significantly enhancing user experience [52,53]. However, these methods often face challenges such as high computational complexity and insufficient real-time processing, particularly when handling large-scale user behavior data, necessitating more efficient algorithm designs.

In summary, GNN-based recommendation algorithms excel in capturing user behavior diversity, higher-order relationship modeling, and complex graph structure representation, offering powerful technical support for the recommendation system domain. Nevertheless, enhancing model interpretability, scalability, and computational efficiency remains a critical challenge and a direction for future advancements in this field.

## Methods

### Methodological framework overview

**Fig 1:**
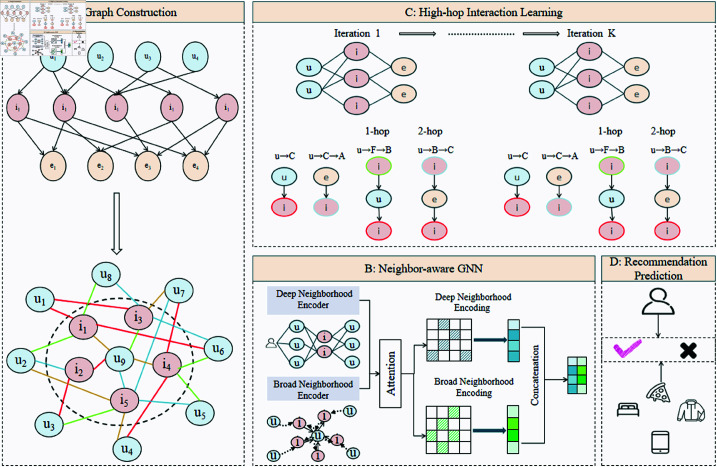
Overall structure diagram of the MBH-GNN model, illustrating the key components: Graph Construction (A), Neighbor-aware GNN (B), High-hop Interaction Learning (C), and Recommendation Prediction (D). The diagram provides a high-level overview of the model architecture, aiding understanding before the detailed explanation.

This study proposes a novel recommendation model named MBH-GNN, specifically designed to address the challenges of user behavior diversity and the complex relationships between users and items. The overall framework integrates three key modules: multi-behavior modeling, a neighbor-aware mechanism, and high-hop interaction learning. First, a multi-behavior interaction graph is constructed, where diverse user-item interactions (e.g., click, favorite, purchase) are represented as multi-typed edges in a heterogeneous graph, providing a semantically rich, structured input for the model. On this basis, a Neighbor-aware Heterogeneous Graph Neural Network (NAH-GNN) is employed to perform localized modeling on direct neighborhood relationships within the multi-behavior graph. Through dynamically assigning behavior-specific importance weights, this module highlights key interactions that contribute the most to the recommendation results. To further enhance the model’s ability to capture long-range dependencies, a High-hop Interaction Learning module is introduced. This module aggregates multi-hop neighborhood information between users and items, allowing the model to capture distant dependencies and enrich global context representations. The three modules in MBH-GNN work in close coordination to achieve multi-level modeling of user interests and behaviors, from localized direct interactions to global long-range dependencies, significantly improving both recommendation accuracy and diversity. The overall framework of MBH-GNN is illustrated in Fig 1.

### Construction of the multi-behavior interaction graph

In recommendation systems, various types of interactions exist between users and items, such as clicks, favorites, add-to-cart actions, and purchases. These interaction types not only reflect the level of user interest in items but also contain rich semantic information. To fully utilize this diverse interaction data, this study constructs a Multi-Behavior Interaction Graph. This graph is a heterogeneous graph where users and items are represented as two types of nodes, and the various interaction types between them are represented as edges with different types. The multi-behavior interaction graph not only captures the diversity of user behaviors but also serves as a foundational input for the subsequent neighbor-aware and high-hop mechanisms, offering a novel perspective for recommendation modeling.The multi-behavior interaction graph helps mitigate the issue of sparse data by integrating multiple types of interactions, allowing the model to leverage richer signals and enhancing its ability to provide accurate recommendations even with limited data.

Let $U=\{u_{1},u_{2},\ldots ,u_{m}\}$ denote the set of users, $V=\{v_{1},v_{2},\ldots ,v_{n}\}$ denote the set of items, and $E=\{e_{ij}^{k}|u_{i}\in U,v_{j}\in V,k\in \{1,2,\ldots ,K\}\}$ denote the set of edges, where *k* represents the interaction type and *K* is the total number of interaction types. Based on the above definitions, the multi-behavior interaction graph *G* = ( *U*  ∪  *V* , *E* )  is initialized with user and item feature embeddings, which are expressed as:


$$\begin{array}{ccc} h_{u_{i}}^{\left(0\right)}=\text{Emb}(u_{i}),\;h_{v_{j}}^{\left(0\right)}=\text{Emb}(v_{j}), & & \end{array}$$ (1)

where Emb ( ⋅ )  represents the initial embedding function, typically generated via pretraining or random initialization.

To reflect the varying influence of different interaction types, a weight $w_{ij}^{k}$ is assigned to each edge, indicating the importance of interaction type *k* between user $u_{i}$ and item $v_{j}$. The weight is calculated as:


$$\begin{array}{ccc} w_{ij}^{k}=f_{k}(x_{ij}^{k}), & & \end{array}$$ (2)

where $x_{ij}^{k}$ represents the feature vector of interaction type *k*, and $f_{k}(\cdot )$ is a learnable function, such as a multi-layer perceptron (MLP).

The multi-behavior interaction graph can be represented by adjacency matrices $A^{k}$, where each interaction type *k* corresponds to one adjacency matrix, and:


$$\begin{array}{ccc} A^{k}[i,j]=w_{ij}^{k}, & & \end{array}$$ (3)

if there exists an interaction of type *k* between user $u_{i}$ and item $v_{j}$, then $A^{k}[i,j]>0$; otherwise, it is 0.

In the multi-behavior interaction graph, node features are updated by aggregating information across different interaction types. First, for each interaction type *k*, neighborhood aggregation is performed as:


$$\begin{array}{cc} h_{u_{i}}^{\left(k\right)} & =\sigma \left(\underset{v_{j}\in N_{i}^{k}}{\sum }w_{ij}^{k}\cdot W^{k}h_{v_{j}}^{\left(0\right)}\right), \end{array}$$ (4)

$$\begin{array}{cc} h_{v_{j}}^{\left(k\right)} & =\sigma \left(\underset{u_{i}\in N_{j}^{k}}{\sum }w_{ij}^{k}\cdot W^{k}h_{u_{i}}^{\left(0\right)}\right), \end{array}$$ (5)

where $N_{i}^{k}$ denotes the neighborhood of user $u_{i}$ under interaction type *k*, $W^{k}$ is the weight matrix for type *k*, and *σ* ( ⋅ )  is an activation function (e.g., ReLU).

Then, the aggregated information from all interaction types is fused to generate the comprehensive node representations:


$$\begin{array}{cc} h_{u_{i}}^{\left(1\right)} & =\text{Fuse}\left(h_{u_{i}}^{\left(1,1\right)},h_{u_{i}}^{\left(1,2\right)},\ldots ,h_{u_{i}}^{\left(1,K\right)}\right), \end{array}$$ (6)

$$\begin{array}{cc} h_{v_{j}}^{\left(1\right)} & =\text{Fuse}\left(h_{v_{j}}^{\left(1,1\right)},h_{v_{j}}^{\left(1,2\right)},\ldots ,h_{v_{j}}^{\left(1,K\right)}\right), \end{array}$$ (7)

where Fuse ( ⋅ )  is a fusion function, which can be a weighted sum, concatenation, or attention mechanism.

**Fig 2:**
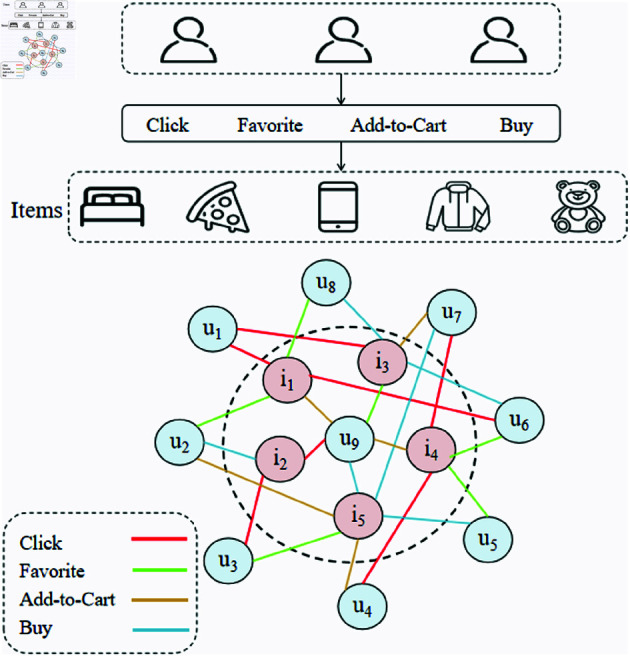
Structure of the multi-behavior interaction graph.

Through these steps, the interactions between users and items are modeled into a semantically rich multi-behavior heterogeneous graph, which provides high expressiveness to capture fine-grained patterns of user behaviors and multi-layered structural information for recommendation systems. The multi-behavior interaction graph is especially effective in addressing the sparsity problem by incorporating multiple interaction types, allowing the model to leverage richer data and improve recommendation performance in scenarios with limited data.The multi-behavior interaction graph plays a pivotal role as the basis for subsequent neighbor-aware and high-hop learning mechanisms. The overall structure of the multi-behavior interaction graph is illustrated in Fig 2.

### Neighbor-aware heterogeneous graph neural network

Based on the multi-behavior interaction graph, this study introduces the NAH-GNN to fully exploit the local interaction relationships between users and items. This method dynamically assigns weights to different types of interactions, capturing their contributions to the recommendation results and generating more precise user and item representations. In the heterogeneous graph, different types of edges represent various interaction behaviors, each with distinct importance. Therefore, the edge weights are adjusted dynamically based on the context of interactions, and an attention mechanism is employed to aggregate neighborhood information, enhancing the expressiveness of user and item embeddings.

**Fig 3:**
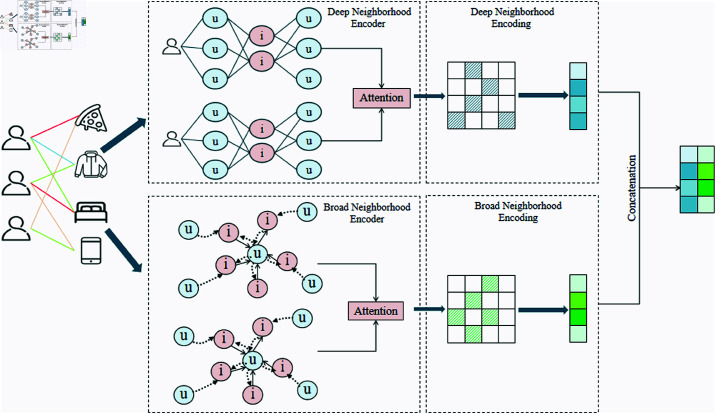
Structure of the neighbor-aware heterogeneous graph neural network.

The overall structure of the Neighbor-aware Heterogeneous Graph Neural Network is shown in Fig 3, where the model dynamically integrates multi-behavior neighborhood information between user and item nodes to improve feature modeling comprehensively.

Let $h_{u_{i}}^{\left(0\right)}$ and $h_{v_{j}}^{\left(0\right)}$ represent the initial embeddings of user $u_{i}$ and item $v_{j}$, respectively. For a specific interaction type k, the embedding of user node $u_{i}$ can be updated using the information from its neighboring item nodes as follows:


$$\begin{array}{ccc} h_{u_{i}}^{\left(k\right)}=\sigma \left(\underset{v_{j}\in N_{i}^{k}}{\sum }\alpha_{ij}^{k}\cdot W^{k}h_{v_{j}}^{\left(0\right)}\right), & & \end{array}$$ (8)

where $N_{i}^{k}$ denotes the neighborhood of user node $u_{i}$ under interaction type k, $W^{k}$ is the linear transformation matrix for interaction type k, and σ(⋅) is an activation function, such as ReLU. The attention score $\alpha_{ij}^{k}$, which indicates the importance of item node $v_{j}$ to user node $u_{i}$ under interaction type k, is defined as:


αijk=exp ⁡  (LeakyReLU (a⊤ ⁡[Wkhui(0)∥Wkhvj(0)]))∑vl∈Nik exp ⁡  (LeakyReLU (a⊤ ⁡[Wkhui(0)∥Wkhvl(0)])), (9)

where a is a learnable parameter for computing attention weights, [⋅∥⋅] denotes vector concatenation, and LeakyReLU is an activation function that introduces non-linearity. Similarly, the embedding of item node $v_{j}$ can be updated using information from its neighboring user nodes:


$$\begin{array}{ccc} h_{v_{j}}^{\left(k\right)}=\sigma \left(\underset{u_{i}\in N_{j}^{k}}{\sum }\alpha_{ji}^{k}\cdot W^{k}h_{u_{i}}^{\left(0\right)}\right), & & \end{array}$$ (10)

where $N_{j}^{k}$ represents the neighborhood of item node $v_{j}$ under interaction type k, and $\alpha_{ji}^{k}$ denotes the corresponding attention score.

To incorporate the influence of all interaction types, the representations of all interaction types are fused to generate the comprehensive node features. The final representation of user node $u_{i}$ is:


$$\begin{array}{ccc} h_{u_{i}}^{\left(1\right)}=\overset{K}{\underset{k=1}{\sum }}\beta_{k}\cdot h_{u_{i}}^{\left(k\right)}, & & \end{array}$$ (11)

and the final representation of item node $v_{j}$ is:


$$\begin{array}{ccc} h_{v_{j}}^{\left(1\right)}=\overset{K}{\underset{k=1}{\sum }}\beta_{k}\cdot h_{v_{j}}^{\left(k\right)}, & & \end{array}$$ (12)

where $\beta_{k}$ is the global weight of interaction type k, which measures the importance of this type in the final node representation and can be calculated through learnable parameters or predefined weights. This approach dynamically captures the characteristics of different interaction types and neighborhood relationships, resulting in more semantically rich user and item embeddings.

By introducing the Neighbor-aware Heterogeneous Graph Neural Network, the model effectively combines the attention mechanism with heterogeneous graph features. It dynamically adjusts the importance of different interaction types and extracts useful contextual information from heterogeneous neighborhoods. This design improves both the accuracy and diversity of recommendation results, especially in complex recommendation scenarios, providing a solid foundation for subsequent optimization and recommendation performance enhancement.

### High-hop interaction learning

In recommendation systems, relying solely on the direct neighborhood relationships between users and items may overlook potential long-range dependencies, which often reveal hidden user interests or implicit associations between items. To address this limitation, this study introduces the High-hop Interaction Learning module, which captures multi-hop neighborhood information to extend the contextual relationship representation between users and items, thereby enhancing the global modeling capability of the recommendation system.

In the multi-behavior interaction graph, the high-order relationships between user and item nodes can be explored through multi-hop paths. Specifically, the high-hop representation of a user node $u_{i}$ is generated by accumulating information from its multi-hop neighbors. In the high-hop mechanism, the feature update at each hop is defined as:


$$\begin{array}{cc} h_{u_{i}}^{\left(l+1\right)} & =\sigma \left(\underset{v_{j}\in N_{i}}{\sum }W^{\left(l\right)}h_{v_{j}}^{\left(l\right)}\right), \end{array}$$ (13)

$$\begin{array}{cc} h_{v_{j}}^{\left(l+1\right)} & =\sigma \left(\underset{u_{i}\in N_{j}}{\sum }W^{\left(l\right)}h_{u_{i}}^{\left(l\right)}\right), \end{array}$$ (14)

where l denotes the hop layer, $N_{i}$ and $N_{j}$ represent the neighborhoods of user $u_{i}$ and item $v_{j}$, respectively, $W^{\left(l\right)}$ is the linear transformation weight for layer l, and σ(⋅) is an activation function (e.g., ReLU). Through iterative updates across multiple layers, the nodes can capture not only direct neighborhood information but also high-hop features from distant neighbors.

However, expanding the number of hop layers can cause the over-smoothing issue, where node features lose their distinctiveness. To address this issue, this study designs a high-hop aggregation mechanism to dynamically adjust the weights of multi-hop information. The final representation of a user node $u_{i}$ is:


$$\begin{array}{cc} h_{u_{i}} & =\overset{L}{\underset{l=0}{\sum }}\gamma^{\left(l\right)}\cdot h_{u_{i}}^{\left(l\right)}, \end{array}$$ (15)

$$\begin{array}{cc} h_{v_{j}} & =\overset{L}{\underset{l=0}{\sum }}\gamma^{\left(l\right)}\cdot h_{v_{j}}^{\left(l\right)}, \end{array}$$ (16)

where L is the maximum hop layer, and $\gamma^{\left(l\right)}$ is the weight for hop l, which can be dynamically learned to optimally distribute the weights across different layers of hop information.

To further enhance the representation of high-hop relationships, an attention-based high-hop information fusion mechanism is introduced. Under this mechanism, the weight of each hop’s neighborhood features is dynamically determined by its relevance to the central node.

The attention-based high-hop aggregation is defined as:


$$\begin{array}{cc} h_{u_{i}} & =\overset{L}{\underset{l=0}{\sum }}\alpha^{\left(l\right)}\cdot h_{u_{i}}^{\left(l\right)}, \end{array}$$ (17)

α(l)= exp ⁡  (LeakyReLU (q⊤ ⁡hui(l))) ∑l′=0L exp ⁡  (LeakyReLU (q⊤ ⁡hui(l′))), (18)

where q is a learnable attention vector used to measure the importance of different hop layers, and $\alpha^{\left(l\right)}$ denotes the attention weight for the l-th hop information.

**Fig 4:**
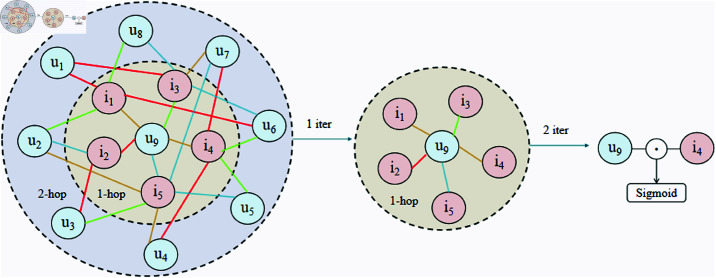
Structure of the high-hop interaction learning module.

This module effectively addresses the limitations of traditional graph neural networks in representing long-range dependencies while avoiding the issues of over-smoothing or feature redundancy caused by excessive layers. By aggregating multi-hop neighborhood information, the model captures deep relationships between users and items from a global perspective, thereby improving the overall recommendation performance. The structure of the High-hop Interaction Learning module is shown in Fig 4 .

## Experiment

### Datasets

This experiment utilizes two publicly available datasets, BeiBei Dataset and Tmall Dataset [54], representing typical scenarios of cross-domain product recommendation and multi-behavior modeling in e-commerce. These datasets contain rich user-item interaction information and detailed metadata about items (e.g., categories, brands), making them highly suitable for studying the application of graph neural networks in recommendation systems and optimizing personalized marketing strategies.

The BeiBei Dataset is a publicly available e-commerce dataset from one of the largest infant product retail sites in China. This dataset contains 21,716 users and 7,977 items, with three types of user-item interactions: click, add-to-cart, and purchase. It provides a comprehensive view of user engagement with various items, including product metadata such as categories and brands. This rich information allows for the creation of a user-item interaction graph, where interactions are represented as edges and item features are used as node features. The BeiBei Dataset is particularly valuable for studying multi-behavior recommendation tasks, as it captures the complexity of user preferences and item relations. The dataset is diverse in terms of user behaviors but may contain biases due to the specific demographic of users, which could influence the results, particularly for niche product categories.

The Tmall Dataset, provided by Alibaba, is a representative multi-behavior dataset in the e-commerce domain. It includes 47,894 users and 99,037 items, with four types of user behaviors: click, add-to-cart, tag-as-favorite, and purchase. This dataset contains millions of interaction records, along with item metadata and timestamps of user behaviors. The Tmall Dataset is highly suitable for constructing multi-behavior heterogeneous graphs, which can be used to explore the relationships between different types of user behaviors. The temporal data in the dataset also allows for capturing the sequential nature of user behaviors, providing valuable insights for optimizing marketing strategies. Similar to the BeiBei dataset, the Tmall dataset may exhibit biases related to over-representation of certain user behaviors or product categories, which can affect model performance, especially in cases where some behaviors are less frequent.

By leveraging these two datasets, we aim to conduct a comprehensive evaluation of graph neural networks’ performance across different scenarios. The BeiBei Dataset emphasizes cross-domain recommendation and the integration of product metadata, while the Tmall Dataset focuses on multi-behavior modeling and temporal dynamics in e-commerce. These datasets serve as a solid foundation for validating the effectiveness of graph neural networks in optimizing personalized marketing strategies. The diversity of user behaviors and the potential biases present in the datasets are important factors that we consider when interpreting the results of the study.

### Data preprocessing

To prepare the datasets for graph neural network modeling and to validate the optimization of personalized marketing strategies, we performed preprocessing on the BeiBei and Tmall Datasets. This included constructing user-item interaction graphs, extracting multi-behavior relationships, and preparing relevant features.

For the BeiBei Dataset, we filtered out users and items with fewer than five interactions to mitigate sparsity. A user-item interaction graph was then created, where users and items were represented as nodes, and interactions (e.g., ratings or reviews) were represented as edges with weights based on normalized rating scores. The normalization of rating scores was performed globally across all users using min-max normalization, scaling the interaction scores between 0 and 1. Item metadata, such as categories and brands, were encoded using one-hot encoding or embeddings and added as node features.

For the Tmall Dataset, which has multi-behavior interactions, we categorized the interactions into clicks, favorites, add-to-cart actions, and purchases. Each behavior was modeled as a distinct edge type in a multi-behavior heterogeneous graph. Item metadata and interaction timestamps were used to further enrich the graph, capturing the sequential nature of user behaviors. We incorporated interaction timestamps as a feature within the graph, enabling the model to capture the sequence and recency of user interactions. The dataset was split chronologically into training, validation, and testing sets to preserve temporal dynamics.

For both datasets, item metadata and interaction features were standardized to ensure uniform scaling. Item metadata were standardized by subtracting the mean and dividing by the standard deviation of each feature. Interaction features were similarly scaled to ensure consistency.

To handle missing or noisy data, we used imputation techniques for missing values and removed outliers based on interaction frequency. This ensured that the data used for training the model was clean and reliable.

These preprocessing steps resulted in graph structures that were suitable for graph neural network modeling. The BeiBei Dataset was transformed into a weighted bipartite graph, while the Tmall Dataset became a multi-behavior heterogeneous graph. These representations effectively capture complex user-item relationships, as well as the temporal and behavioral diversity in the datasets.

### Experimental setup

This experiment was conducted on the BeiBei Dataset and Tmall Dataset to train and evaluate the proposed model. The experimental environment and hyperparameter settings were standardized to ensure fairness and reproducibility. The overall training process is outlined in Algorithm 1.

**Algorithm 1 Training process of graph neural network (MBH-GNN).**



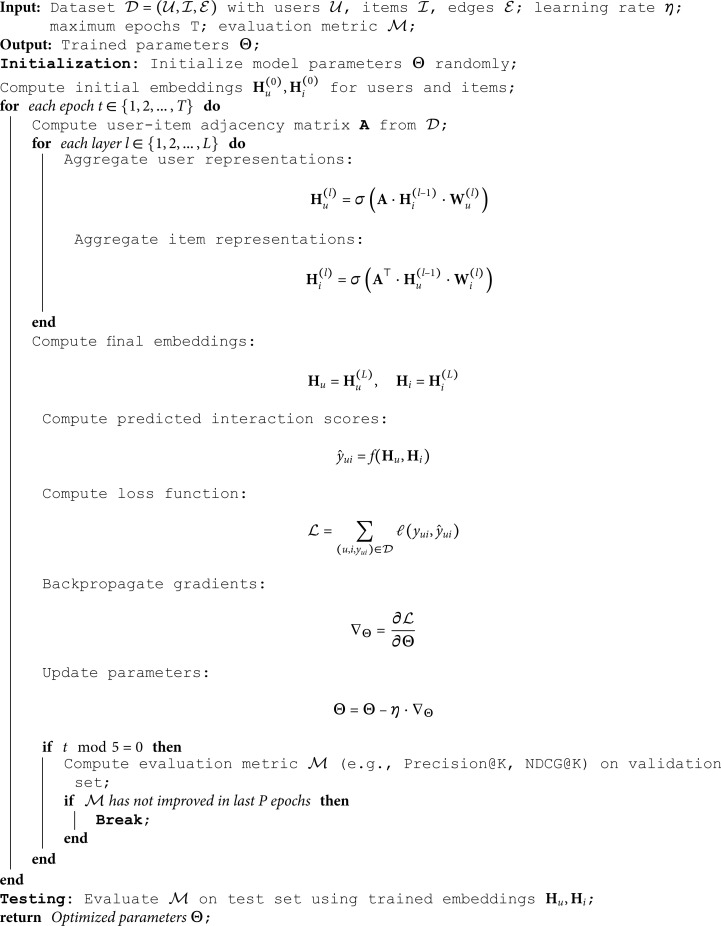



The experiments were performed on a high-performance server equipped with an NVIDIA Tesla V100 GPU (32 GB memory) and an Intel Xeon Gold 6248R CPU. The operating system was Ubuntu 20.04. The model was implemented using the PyTorch framework in combination with the Deep Graph Library (DGL) for graph neural network operations.

In all experiments, the embedding size for user and item nodes was set to 128. The embedding size of 128 was chosen after experimenting with different sizes, where we observed that it provided a good balance between model performance and computational efficiency, without leading to overfitting or excessive computational cost. The graph neural network consisted of two graph convolutional layers, each followed by a ReLU activation function to enhance non-linear representation capabilities. Dropout was applied after each layer with a dropout rate of 0.5 to prevent overfitting. The Adam optimizer was used for parameter optimization with an initial learning rate of 0.001, which was selected based on cross-validation experiments to optimize performance. The batch size was set to 512, determined through experimentation to balance memory usage and training efficiency. Early stopping was employed during training, terminating the process when the performance on the validation set did not improve for 5 consecutive epochs, with the maximum number of epochs set to 50.

During training, user and item embeddings were updated through the graph neural network by aggregating features from their respective neighbors. For the Tmall Dataset, the multi-behavior characteristics were modeled as different edge types in a heterogeneous graph, where shared weights were used to integrate the multi-behavior information. Additionally, high-hop information was used as an enhancement mechanism to expand the neighborhood scope during embedding propagation, capturing complex relationships between users and items.

The experimental environment and parameter configurations were designed to ensure efficient model training while capturing the multi-level relationships between users and items. This setup provides a robust foundation for analyzing experimental results and supports the further introduction of evaluation metrics in subsequent sections.

## Model evaluation

To comprehensively evaluate the performance of the proposed model in the recommendation system, we employ classic metrics including HR (Hit Ratio), Precision, Recall, and NDCG (Normalized Discounted Cumulative Gain). These metrics effectively measure the model’s recommendation quality and its potential to optimize personalized marketing strategies. The choice of HR@10 and NDCG@10 is particularly relevant as they focus on evaluating the model’s performance in the top-K recommendations, which is a key factor in personalized recommendation systems. HR@10 assesses whether relevant items appear in the top 10 recommendations, while NDCG@10 evaluates both the relevance and ranking quality of those recommendations. In addition to HR@10 and NDCG@10, experiments were also conducted with K = 5, K = 20, and K = 50 to further assess the model’s performance across different recommendation list lengths. This provides a more comprehensive evaluation of the model’s generalizability and robustness across various application scenarios.

HR measures whether the user’s actual interacted items appear in the recommendation list. It is a binary metric that evaluates the hit rate of recommendations, defined as:


$$HR@K=\frac{\text{Number of Hits}}{\text{Number of Users}}$$

A hit is recorded if the user’s interacted items are found in the top-K recommended list.

Precision measures the proportion of relevant items in the recommendation list, defined as:


$$Precision@K=\frac{\underset{u\in U}{\sum }|\text{Relevant Items in Top-K}|}{K\cdot |U|}$$

Higher precision indicates that the items in the recommendation list are more aligned with the user’s interests.

Recall measures the proportion of relevant items in the recommendation list out of all items the user has interacted with, defined as:


$$Recall@K=\frac{\underset{u\in U}{\sum }|\text{Relevant Items in Top-K}|}{\left|\text{Relevant Items for User}\right|}$$

Higher recall indicates the recommendation system’s ability to cover a larger portion of the user’s historical interactions.

NDCG evaluates the ranking quality of the recommendation list. It is defined as:


NDCG@K=∑i=1Kreli log ⁡ 2(i+1)IDCG@K

where ${\text{rel}}_{i}$ represents the relevance of the item at rank i, and IDCG@K is the ideal DCG value for the top-K ranking. NDCG measures how well the recommended items are ranked according to the user’s preferences.

During the experiment, model parameters were fine-tuned using the validation set, and the final evaluation was conducted on the test set. By calculating HR, Precision, Recall, and NDCG, we can comprehensively assess the model’s performance in the recommendation task and its contribution to optimizing personalized marketing strategies. The results highlight the model’s strengths in recommendation quality, ranking capability, and accurately capturing user interests.

## Baseline models

To validate the effectiveness of the proposed model, we compare it with seven state-of-the-art baseline models, each leveraging different techniques in recommendation systems and graph-based learning:

- NCF (Neural Collaborative Filtering): A neural network-based collaborative filtering model that replaces traditional matrix factorization with non-linear neural architectures to model user-item interactions.
- NGCF (Neural Graph Collaborative Filtering): A graph-based collaborative filtering model that leverages high-order connectivity in user-item interaction graphs to propagate embeddings.
- TAG (Triple-Hierarchical Attention Graph) [55]: A framework that combines triple-hierarchical attention with graph-based learning to extract features from user reviews and social relationships for rating prediction.
- CompGCN (Composition-based Graph Convolutional Network) [56]: A graph convolutional network for multi-relational graphs that jointly embeds both nodes and relationships using entity-relation composition operators.
- TrustTF [57]: A tensor factorization model that incorporates user trust and implicit feedback to alleviate data sparsity and improve context-aware recommendation accuracy.
- CombiGCN [58]: A GCN-based recommendation framework that combines user-user and user-item interaction graphs, leveraging collaborative signals with lightweight graph convolution and linear propagation.
- INFLECT-DGNN [59]: A profit-driven dynamic graph neural network that integrates temporal dependencies and network representation for influencer prediction in referral and targeted marketing.

These baseline models are selected to provide a comprehensive evaluation, covering collaborative filtering, multi-relational graph modeling, social information integration, and dynamic graph learning. By comparing the proposed model with these approaches, we aim to highlight its advantages in personalized marketing and recommendation tasks.

## Experimental results and analysis

### Performance comparison with baseline models.

Table 1 presents the performance comparison between the proposed model (MBH-GNN) and multiple baseline models on the BeiBei and Tmall datasets. Overall, MBH-GNN achieved the best performance across all evaluation metrics on both datasets, demonstrating its effectiveness in recommendation tasks. On the BeiBei dataset, MBH-GNN achieved an HR@10 of 0.789 and a Recall@10 of 0.383, significantly outperforming other baseline models. For instance, compared to the next-best model, CombiGCN, MBH-GNN achieved a 0.028 improvement in HR@10 and a 0.023 improvement in Recall@10, highlighting its ability to capture complex user-item interactions effectively. Similarly, on the Tmall dataset, MBH-GNN achieved an HR@10 of 0.773 and a Recall@10 of 0.375, representing a 0.028 and 0.023 improvement, respectively, over CombiGCN. Although the overall performance on the Tmall dataset is slightly lower than on the BeiBei dataset, MBH-GNN consistently demonstrates its superiority over baseline models. The higher performance on the BeiBei dataset may be attributed to its denser interaction records. In contrast, the Tmall dataset contains more diverse user behaviors, which may present challenges in behavior modeling. However, MBH-GNN still maintains strong performance on the Tmall dataset, particularly in ranking quality. In summary, the experimental results demonstrate that MBH-GNN not only performs exceptionally on individual datasets but also maintains consistent superior performance across different datasets, showcasing its generalizability and adaptability in recommendation tasks. Furthermore, the performance differences between datasets highlight MBH-GNN’s robustness and effectiveness in handling complex user behaviors and sparse data scenarios.

**Table 1:** Performance comparison on BeiBei and Tmall datasets.

Model	BeiBei Dataset				Tmall Dataset			
	HR@10	Precision@10	Recall@10	NDCG@10	HR@10	Precision@10	Recall@10	NDCG@10
NCF	0.652	0.172	0.294	0.243	0.637	0.169	0.289	0.231
NGCF	0.721	0.192	0.335	0.282	0.706	0.187	0.327	0.270
TAG	0.742	0.198	0.349	0.296	0.726	0.193	0.342	0.285
CompGCN	0.711	0.186	0.320	0.275	0.689	0.181	0.312	0.262
TrustTF	0.732	0.191	0.344	0.287	0.714	0.188	0.338	0.276
CombiGCN	0.761	0.204	0.360	0.312	0.745	0.198	0.352	0.302
INFLECT-DGNN	0.749	0.201	0.355	0.305	0.732	0.195	0.347	0.295
MBH-GNN	0.789	0.216	0.383	0.330	0.773	0.211	0.375	0.319

The results in the Table 2 show the performance comparison of various models on the BeiBei and Tmall datasets, evaluated using NDCG and HR at different K values. MBH-GNN consistently outperforms the other models across all K values, including K = 5, K = 10, K = 20, and K = 50, in both HR and NDCG metrics. This indicates that MBH-GNN is highly effective in ranking relevant items, especially as the recommendation list length increases. On the BeiBei dataset, the MBH-GNN model achieves the highest HR of 0.789 at K = 10 and 0.812 at K = 50, which are significantly higher than those of other models such as NCF and NGCF. The NDCG scores show a similar trend, with MBH-GNN outperforming other models at all K values. For example, the model’s NDCG@10 reaches 0.330, and at K=50, it improves to 0.351, further demonstrating its ability to effectively rank relevant items in the top positions. The overall performance improves as the K value increases, which suggests that MBH-GNN provides better recommendations when more items are considered, thus making it suitable for a wider range of application scenarios. On the Tmall dataset, MBH-GNN again delivers the best performance across both HR and NDCG metrics. At K = 10, it achieves an HR of 0.773 and at K = 50, an HR of 0.795, outperforming other models like NCF and TAG. The NDCG scores follow a similar trend, with MBH-GNN achieving an NDCG@10 of 0.319 and an NDCG@50 of 0.341. These results further validate that MBH-GNN excels at capturing complex user-item interactions, providing highly relevant recommendations at the top ranks, and scaling well as the number of recommended items increases. These comprehensive results highlight that MBH-GNN not only provides high-quality recommendations but also performs well across different lengths of recommendation lists. Compared to traditional models like NCF and NGCF, MBH-GNN shows superior generalization and robustness, demonstrating its capability to deliver accurate and relevant recommendations in diverse scenarios and datasets.

**Table 2:** Comparison of model performance on the BeiBei and Tmall datasets, evaluated using multiple recommendation metrics: NDCG and HR at different K values.

Model	BeiBei Dataset							
	NDCG@5	HR@5	NDCG@10	HR@10	NDCG@20	HR@20	NDCG@50	HR@50
NCF	0.213	0.607	0.243	0.652	0.261	0.683	0.271	0.694
NGCF	0.231	0.623	0.282	0.721	0.291	0.732	0.301	0.743
TAG	0.241	0.634	0.296	0.742	0.306	0.753	0.316	0.764
CompGCN	0.221	0.615	0.275	0.711	0.286	0.723	0.296	0.734
TrustTF	0.236	0.628	0.287	0.732	0.297	0.738	0.307	0.749
CombiGCN	0.253	0.647	0.312	0.761	0.323	0.772	0.333	0.783
INFLECT-DGNN	0.246	0.638	0.305	0.749	0.316	0.758	0.326	0.769
MBH-GNN	0.262	0.653	0.330	0.789	0.341	0.801	0.351	0.812
**Model**	**Tmall Dataset**							
	**NDCG@5**	**HR@5**	**NDCG@10**	**HR@10**	**NDCG@20**	**HR@20**	**NDCG@50**	**HR@50**
NCF	0.202	0.593	0.231	0.637	0.241	0.652	0.251	0.663
NGCF	0.222	0.613	0.270	0.706	0.281	0.723	0.291	0.734
TAG	0.233	0.624	0.285	0.726	0.296	0.737	0.306	0.748
CompGCN	0.212	0.604	0.262	0.689	0.273	0.703	0.283	0.714
TrustTF	0.227	0.618	0.276	0.714	0.287	0.728	0.297	0.739
CombiGCN	0.243	0.635	0.302	0.745	0.313	0.758	0.323	0.769
INFLECT-DGNN	0.237	0.629	0.295	0.732	0.306	0.743	0.316	0.754
MBH-GNN	0.253	0.647	0.319	0.773	0.331	0.784	0.341	0.795

### Performance analysis in sparse and cold start scenarios.

**Fig 5:**
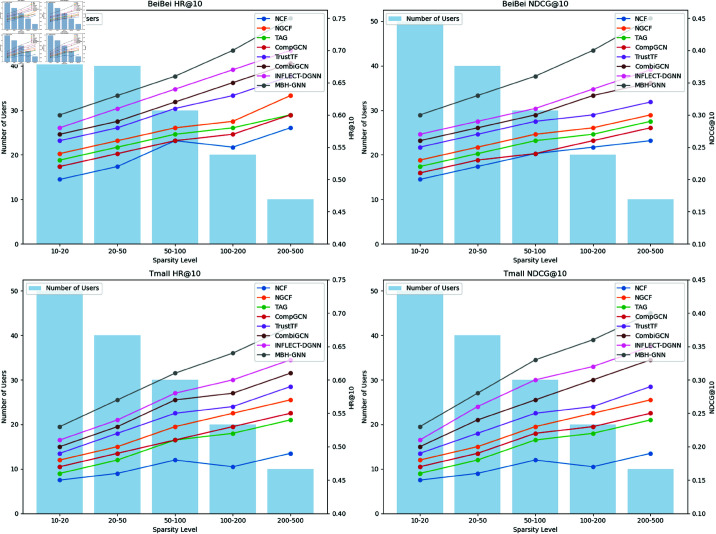
The comparison of Hit Rate (HR@10) and Normalized Discounted Cumulative Gain (NDCG@10) across varying data sparsity levels on BeiBei and Tmall datasets.

Fig 5 illustrates the performance of different models across varying data sparsity levels, highlighting the ability of each model to handle sparse data scenarios. The results demonstrate that MBH-GNN consistently outperforms other models in all sparsity intervals, particularly in high sparsity intervals (e.g., 100-200 and 200-500), where its HR@10 and NDCG@10 metrics show significant improvements. This indicates that MBH-GNN is capable of capturing the complex relationships between users and items, maintaining stability and high accuracy even in sparse data environments. From the trends observed, other models exhibit varying degrees of sensitivity to sparsity changes. For instance, TAG and TrustTF show competitive performance in low sparsity intervals (e.g., 10-20 and 20-50), but their performance fluctuates significantly as sparsity increases. In contrast, CombiGCN and INFLECT-DGNN exhibit more stable performance in medium sparsity intervals (e.g., 50-100 and 100-200), but their overall performance is still lower than that of MBH-GNN. These results suggest that while these models can capture some user interaction patterns, they face limitations in leveraging potential information under highly sparse conditions. Notably, MBH-GNN demonstrates a smooth and stable performance improvement across all sparsity intervals, highlighting its robustness and generalizability under varying sparsity conditions. In particular, while the performance of most other models deteriorates significantly in high sparsity conditions, MBH-GNN remains robust and leads in performance. This further validates the model’s adaptability and effectiveness in addressing data sparsity issues, showcasing its strong potential for practical recommender system applications. As shown in the figure, MBH-GNN’s design effectively mitigates sparsity challenges and achieves superior recommendation accuracy compared to other methods.

**Fig 6:**
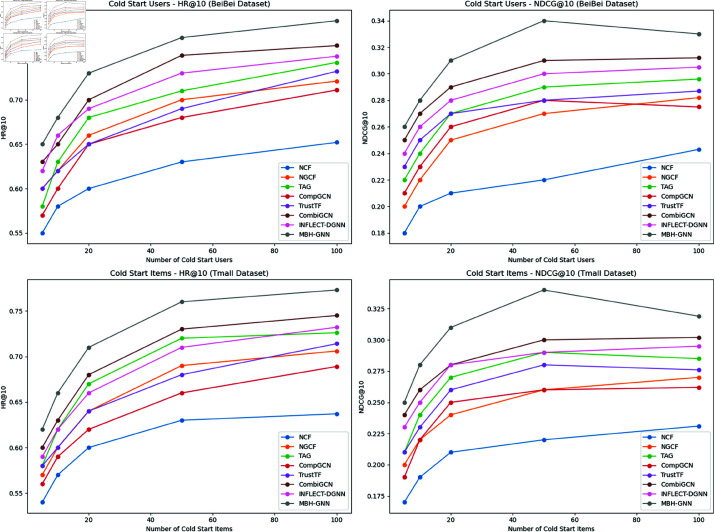
Performance of various models in cold start scenarios on BeiBei and Tmall datasets. The horizontal axis represents the number of cold start users/items, while the vertical axis shows the respective performance metrics.

Fig 6 demonstrates the performance of various models in cold start scenarios, focusing on both user and item cold starts across the BeiBei and Tmall datasets. The results highlight how the models adapt to increasing numbers of cold start users or items, with HR@10 and NDCG@10 serving as key evaluation metrics. From the subfigures, it is evident that MBH-GNN maintains a clear advantage over baseline models, particularly in scenarios with fewer cold start users or items, where data sparsity is more severe. For cold start users on the BeiBei dataset, MBH-GNN demonstrates a robust growth trend in HR@10 and NDCG@10 as the number of users increases. This indicates that MBH-GNN effectively captures sparse user-item interactions and provides higher quality recommendations compared to other models. In contrast, baseline models such as NCF and NGCF show slower improvement, reflecting their limited ability to adapt to sparse scenarios. In the Tmall dataset’s cold start item scenarios, a similar pattern is observed. MBH-GNN outperforms all other models across both HR@10 and NDCG@10 metrics, particularly in high sparsity conditions. While other models such as CombiGCN and INFLECT-DGNN exhibit stable performance, they lag behind MBH-GNN, which highlights its superior ability to model item-related interactions in sparse data settings. Overall, these results validate MBH-GNN’s robustness and adaptability in addressing cold start challenges, making it a promising solution for real-world recommendation systems that face user and item sparsity issues.

### Multi-behavior interaction analysis.

**Fig 7:**
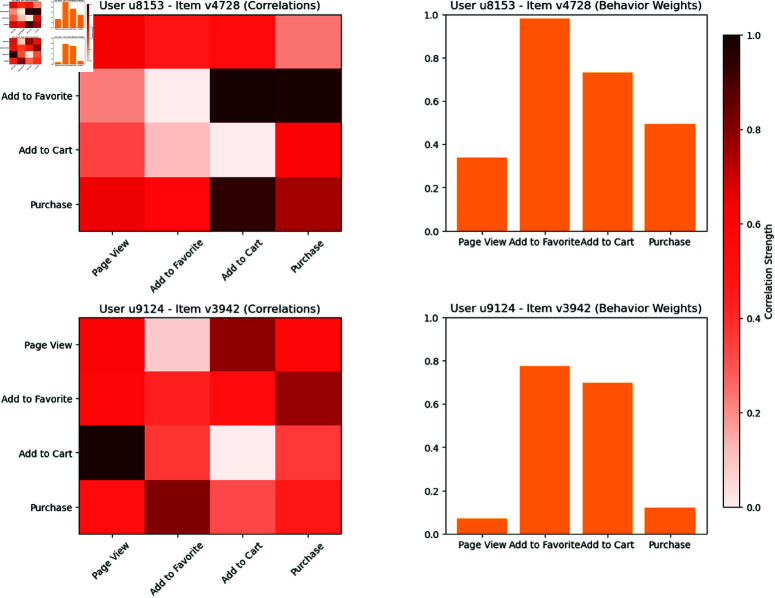
Visualization of user-item multi-behavior interactions in MBH-GNN. Heatmaps represent the correlation strength between different behaviors, and bar charts show the relative weights of each behavior. Higher correlations and weights indicate greater influence on recommendation decisions.

Fig 7 provides an in-depth visualization of the multi-behavior interaction modeling in MBH-GNN, showcasing how the model captures and utilizes user-item relationships for improved recommendation performance. From the heatmaps, it is evident that certain behaviors, such as "Add to Cart" and "Purchase," exhibit stronger correlations compared to "Page View" or "Add to Favorite." This indicates that these behaviors are more closely linked and play a significant role in the recommendation process. The intensity of the colors highlights the varying degrees of interdependence among behaviors, which MBH-GNN effectively learns to enhance recommendation quality. The bar charts provide additional insights into the relative importance of each behavior. For both user-item pairs, "Purchase" consistently receives the highest weight, reflecting its critical role in determining user preferences. However, other behaviors, such as "Add to Cart," also contribute significantly, demonstrating the model’s ability to balance multiple behavior signals. Overall, this figure emphasizes the strength of MBH-GNN in leveraging behavior interdependencies and assigning appropriate weights to different behaviors. These insights highlight the model’s ability to produce highly personalized and accurate recommendations by effectively integrating multi-behavior signals.

### Ablation study.

Table 3 presents the ablation study results for MBH-GNN, where key modules, including Multi-Behavior Modeling (MB), High-Hop Aggregation (HH), Graph Neural Network Layers (GNN), and Attention Mechanism (Attn), were removed to evaluate their contributions to the overall model performance. The results clearly show that removing any module leads to a performance drop across all evaluation metrics, which demonstrates the importance of each component in the MBH-GNN architecture. Removing the MB module results in a significant decline in performance on both datasets. Specifically, HR@10 drops from 0.789 and 0.773 to 0.763 and 0.745 on the BeiBei and Tmall datasets, respectively, while Recall@10 decreases from 0.383 and 0.375 to 0.360 and 0.352. This drop occurs because the model loses the ability to capture diverse user behaviors, which affects recommendation accuracy. The removal of the HH module leads to further performance degradation, indicating that capturing higher-order relationships between users and items is necessary for improving recommendation quality. Under this configuration, HR@10 drops to 0.752 and 0.733, while Recall@10 drops to 0.348 and 0.340 on the BeiBei and Tmall datasets, respectively. Without the HH module, the model is unable to capture long-range user-item interactions, which affects its performance. The most significant performance decline occurs when the GNN layers are removed (w/o GNN), particularly in the NDCG metric, which drops from 0.330 and 0.319 to 0.290 and 0.278 on the BeiBei and Tmall datasets, respectively. Without the GNN layers, the model cannot effectively integrate neighborhood information, leading to substantial performance deterioration. The removal of the Attn module has a relatively smaller impact on overall performance, but it still results in noticeable declines in ranking-related metrics. For instance, NDCG@10 drops from 0.330 and 0.319 to 0.308 and 0.294 on the BeiBei and Tmall datasets, respectively. This indicates that the Attn module’s ability to adjust the ranking is important for overall recommendation quality. Overall, the ablation study results demonstrate that the performance of MBH-GNN relies on the collaborative contributions of its core modules: MB, HH, GNN, and Attn. Each module contributes to different aspects of the recommendation process, enhancing both accuracy and ranking quality.

**Table 3:** Ablation study results on BeiBei and Tmall datasets.

Model	BeiBei Dataset				Tmall Dataset			
	HR@10	Precision@10	Recall@10	NDCG@10	HR@10	Precision@10	Recall@10	NDCG@10
Full MBH-GNN	0.789	0.216	0.383	0.330	0.773	0.211	0.375	0.319
w/o MB	0.763	0.204	0.360	0.312	0.745	0.198	0.352	0.302
w/o HH	0.752	0.198	0.348	0.300	0.733	0.191	0.340	0.287
w/o GNN	0.741	0.193	0.342	0.290	0.720	0.186	0.330	0.278
w/o Attn	0.757	0.201	0.354	0.308	0.739	0.194	0.345	0.294

Table 4 clearly demonstrate the importance of incorporating multi-hop neighborhood information for improving recommendation performance. On the BeiBei dataset, the HR@10 increases from 0.722 to 0.789 as the number of hops extends from 1-hop to 1+2+3-hop. Similarly, the NDCG@10 rises from 0.282 to 0.330. This trend indicates that the additional information from 2-hop and 3-hop neighbors significantly enhances the model’s ability to capture deeper user-item relationships and refine recommendation rankings. A similar trend is observed on the Tmall dataset, where HR@10 improves from 0.702 (1-hop) to 0.773 (1+2+3-hop), and NDCG@10 increases from 0.270 to 0.319. The consistent improvement across both datasets highlights the generalizability of the multi-hop neighborhood information. Notably, the performance gains between 1+2-hop and 1+2+3-hop are smaller compared to those between 1-hop and 1+2-hop. This suggests that while additional hops contribute valuable information, diminishing returns may occur as the neighborhood radius expands further, possibly due to the inclusion of less relevant nodes or increased noise. Overall, the results validate the effectiveness of MBH-GNN in leveraging multi-hop neighborhood information to improve recommendation quality. This analysis underscores the importance of modeling higher-order user-item interactions in graph-based recommendation systems.

**Table 4:** Neighborhood contribution analysis on BeiBei and Tmall datasets. This table illustrates the impact of different neighborhood hops (1-hop, 1+2-hop, 1+2+3-hop) on HR@10 and NDCG@10 for both datasets.

Hop	BeiBei Dataset		Tmall Dataset	
	HR@10	NDCG@10	HR@10	NDCG@10
1-hop	0.722	0.282	0.702	0.270
1+2-hop	0.764	0.312	0.745	0.302
1+2+3-hop	0.789	0.330	0.773	0.319

## Conclusion

In this work, we proposed the MBH-GNN model, which innovatively integrates neighborhood awareness, multi-behavior interaction modeling, and high-order hop relation learning to significantly enhance personalized recommendation performance. Specifically, the model utilizes multi-behavior interaction graph modeling to capture the semantic relationships between various user-item interaction types. Through the neighborhood-aware heterogeneous graph neural network (NAH-GNN), the model dynamically assigns weights to different interaction behaviors in local neighborhood modeling, generating more precise embeddings for users and items. Additionally, the high-order hop relation learning mechanism further captures long-range dependencies, improving the expressive power of contextual modeling. Experimental results on BeiBei and Tmall datasets demonstrate that MBH-GNN outperforms existing baseline models across multiple evaluation metrics while showing robustness in sparse data and cold start scenarios.

However, MBH-GNN has certain limitations. On the one hand, the computational complexity increases with deeper hop relations, which may limit its application on large-scale datasets. On the other hand, while the multi-behavior interaction modeling enhances recommendation accuracy, the current weight assignment mechanism may introduce bias towards rare behaviors, potentially affecting recommendation diversity.

In the future, we plan to optimize the model’s efficiency by employing lightweight sampling strategies or graph computation frameworks to reduce computational overhead. Additionally, we aim to explore more flexible behavior modeling mechanisms to dynamically adapt to the complexity and diversity of user behaviors. In summary, this work offers fresh perspectives on fine-grained modeling in multi-behavior recommendation systems and showcases the potential of graph neural networks in capturing higher-order relationships, providing both theoretical and practical solutions to tackle data sparsity and cold start challenges in recommender systems.

## References

[1] Platform KNIME Analytics. Internet. Zurich, Switzerland: KNIME AG; 2020.

[2] Acuti D, Vocino A, Mazzoli V, Donvito R. The effects of QR delivered content on perceived product value. J Strateg Mark. 2022;30(5):510–32.

[3] Sharma R, Shaikh A, Li E. Designing recommendation or suggestion systems: looking to the future. Electron Mark. 2021;31:243–52.

[4] Hu F, Wu F, Gu H, Abbas G, Alanazi MD, Othmen S, et al. Transforming agriculture with advanced robotic decision systems via deep recurrent learning. Exp Syst Appl. 2025;259:125123. doi: 10.1016/j.eswa.2024.125123

[5] Pleskach V, Bulgakova O, Zosimov V, Vashchilina E, Tumasoniene I. An E-commerce recommendation system based on analysis of consumer behavior models. Proc Int Solut Conf. 2023:210–21.

[6] Addagarla S, Amalanathan A. A survey on comprehensive trends in recommendation systems & applications. Int J Electron Commer Stud. 2019;10(1):65–88.

[7] Xu K, Zhou H, Zheng H, Zhu M, Xin Q. Intelligent classification and personalized recommendation of e-commerce products based on machine learning. arXiv preprint 2024

[8] Ko H, Lee S, Park Y, Choi A. A survey of recommendation systems: recommendation models, techniques, and application fields. Electronics 2022;11(1):141. doi: 10.3390/electronics11010141

[9] Zhang W, Liu F, Xu D, Jiang L. Recommendation system in social networks with topical attention and probabilistic matrix factorization. PLoS One 2019;14(10):e0223967. doi: 10.1371/journal.pone.0223967 31671119 PMC6822766

[10] Ahmadkhani S, Moghaddam ME. A social image recommendation system based on deep reinforcement learning. PLoS One 2024;19(4):e0300059. doi: 10.1371/journal.pone.0300059 38574062 PMC10994284

[11] Koren Y, Rendle S, Bell R. Advances in collaborative filtering. Recomm Syst Handb. 2021:91–142.

[12] Zheng L, Lu CT, Jiang F, Zhang J, Yu PS. Spectral collaborative filtering. In: Proceedings of the 12th ACM Conference on Recommender Systems. 2018. p. 311–9.

[13] Chen C, Zhang M, Zhang Y, Liu Y, Ma S. Efficient neural matrix factorization without sampling for recommendation. ACM Trans Inf Syst 2020;38(2):1–28. doi: 10.1145/3373807

[14] Yuan X, Han L, Qian S, Zhu L, Zhu J, Yan H. Preliminary data-based matrix factorization approach for recommendation. Inf Process Manag. 2021:58(1);102384.

[15] Perifanis V, Efraimidis PS. Federated neural collaborative filtering. Knowl-Based Syst. 2022;242:108441. doi: 10.1016/j.knosys.2022.108441

[16] Bobadilla J, Gutiérrez A, Alonso S, González-Prieto A. Neural collaborative filtering classification model to obtain prediction reliabilities. arXiv preprint 2024

[17] Wu F, Wu L, Liu S, Abbas G, Othmen S, Wang J. Regulating learning module for patient monitoring interactive event detecting robots. Exp Syst Appl. 2025;260:125383. doi: 10.1016/j.eswa.2024.125383

[18] Nassar N, Jafar A, Rahhal Y. A novel deep multi-criteria collaborative filtering model for recommendation system. Knowl-Based Syst. 2020;187:104811. doi: 10.1016/j.knosys.2019.06.019

[19] Wu S, Sun F, Zhang W, Xie X, Cui B. Graph neural networks in recommender systems: a survey. ACM Comput Surv 2022;55(5):1–37. doi: 10.1145/3535101

[20] Xu Z, Wang J, Hu F, Abbas G, Touti E, Albekairi M, et al. Improved camouflaged detection in the large-scale images and videos with minimum boundary contrast in detection technique. Exp Syst Appl. 2024;249:123558. doi: 10.1016/j.eswa.2024.123558

[21] Wang J, Hu F, Abbas G, Albekairi M, Rashid N. Enhancing image categorization with the quantized object recognition model in surveillance systems. Exp Syst Appl. 2024;238:122240. doi: 10.1016/j.eswa.2023.122240

[22] Gurukar S, Pancha N, Zhai A, Kim E, Hu S, Parthasarathy S, et al. Multibisage: a web-scale recommendation system using multiple bipartite graphs at Pinterest. arXiv preprint. 2022. https://arxiv.org/abs/2205.10666

[23] Sun W, Chang K, Zhang L, Meng K. INGCF: An improved recommendation algorithm based on NGCF. In: Proceedings of the International Conference on Algorithms Archit Parallel Process. 2021:116–29.

[24] Ye F, Lu X, Li H, Chen Z. Transfer learning from rating prediction to Top-k recommendation. PLoS One 2024;19(3):e0300240. doi: 10.1371/journal.pone.0300240 38547150 PMC10977712

[25] T R M, Vinoth Kumar V, Lim S-J. UsCoTc: improved collaborative filtering (CFL) recommendation methodology using user confidence, time context with impact factors for performance enhancement. PLoS One 2023;18(3):e0282904. doi: 10.1371/journal.pone.0282904 36921014 PMC10016635

[26] DeMatteo C, Jakubowski J, Stazyk K, Randall S, Perrotta S, Zhang R. The headaches of developing a concussion app for youth. Int J E-Health Med Commun 2024;15(1):1–20. doi: 10.4018/ijehmc.352514

[27] Almayyan WI, AlGhannam BA. Detection of kidney diseases. Int J E-Health Med Commun 2024;15(1):1–21. doi: 10.4018/ijehmc.354587

[28] Wang M. Applying Internet information technology combined with deep learning to tourism collaborative recommendation system. PLoS One 2020;15(12):e0240656. doi: 10.1371/journal.pone.0240656 33271589 PMC7714558

[29] Bobadilla J, Alonso S, Hernando A. Deep learning architecture for collaborative filtering recommender systems. Appl Sci. 2020:10(7);2441.

[30] Javed U, Shaukat K, Hameed I, Iqbal F, Alam T, Luo S. A review of content-based and context-based recommendation systems. Int J Emerg Technol Learn. 2021;16(3):274–306.

[31] Colace F, Conte D, De Santo M, Lombardi M, Santaniello D, Valentino C. A content-based recommendation approach based on singular value decomposition. Connect Sci. 2022;34(1):2158–76.

[32] Wang J, Li F, Lv S, He L, Shen C. Physically realizable adversarial creating attack against vision-based BEV space 3D object detection. IEEE Trans Image Process. 2025. 10.1109/TIP.2025.3526056 40030990

[33] Wang J, Li F, He L. A unified framework for adversarial patch attacks against visual 3D object detection in autonomous driving. IEEE Trans Circuits Syst Video Technol. 2025.

[34] Van Dat N, Van Toan P, Thanh T. Solving distribution problems in content-based recommendation system with Gaussian mixture model. Appl Intell. 2022;52(2):1602–14.

[35] Xu W, Xie Q, Yang S, Cao J, Pang S. Enhancing content-based recommendation via large language model. In: Proceedings of the 33rd ACM International Conference on Information and Knowledge Management. 2024:4153–7.

[36] Di Rocco J, Di Sipio C, Di Ruscio D, Nguyen P. A GNN-based recommender system to assist the specification of metamodels and models. In: Proceedings of the ACM/IEEE 24th International Conference on Model Driven Engineering Languages and Systems (MODELS). 2021:70–81.

[37] Da’u A, Salim N. Recommendation system based on deep learning methods: a systematic review and new directions. Artif Intell Rev. 2020;53(4):2709–48.

[38] Khanal S, Prasad P, Alsadoon A, Maag A. A systematic review: machine learning based recommendation systems for e-learning. Educ Inf Technol. 2020;25(4):2635–64.

[39] Gao C, Zheng Y, Li N, Li Y, Qin Y, Piao J, et al. A survey of graph neural networks for recommender systems: challenges, methods, and directions. ACM Trans Recomm Syst 2023;1(1):1–51. doi: 10.1145/3568022

[40] Ren B, Wang Z. Strategic focus, tasks, and pathways for promoting China’s modernization through new productive forces. J Xi’an Univ Finance Econ. 2024;1:3–11.

[41] Chang J, Gao C, Zheng Y, Hui Y, Niu Y, Song Y, et al. Sequential recommendation with graph neural networks. In: Proceedings of the 44th International ACM SIGIR Conference on Research and Development in Information Retrieval. 2021. p. 378–87.

[42] Zhang L, Liu J, Wei Y, An D, Ning X. Self-supervised learning-based multi-source spectral fusion for fruit quality evaluation: a case study in mango fruit ripeness prediction. Inf Fusion. 2025;117:102814. doi: 10.1016/j.inffus.2024.102814

[43] Zhang H, Yu L, Wang G, Tian S, Yu Z, Li W, et al. Cross-modal knowledge transfer for 3D point clouds via graph offset prediction. Pattern Recognit. 2025;162:111351. doi: 10.1016/j.patcog.2025.111351

[44] El Alaoui D, Riffi J, Sabri A, Aghoutane B, Yahyaouy A, Tairi H. Deep GraphSAGE-based recommendation system: jumping knowledge connections with ordinal aggregation network. Neural Comput Applic 2022;34(14):11679–90. doi: 10.1007/s00521-022-07059-x

[45] Wang B, Xu H, Li C, Li Y, Wang M. TKGAT: Graph attention network for knowledge-enhanced tag-aware recommendation system. Knowl-Based Syst. 2022;257:109903. doi: 10.1016/j.knosys.2022.109903

[46] Liu K, Xue F, Guo D, Wu L, Li S, Hong R. MEGCF: multimodal entity graph collaborative filtering for personalized recommendation. ACM Trans Inf Syst 2023;41(2):1–27. doi: 10.1145/3544106

[47] Huang T, Dong Y, Ding M, Yang Z, Feng W, Wang X, et al. Mixgcf: an improved training method for graph neural network-based recommender systems. In: Proceedings of the 27th ACM SIGKDD Conference on Knowledge Discovery and Data Mining. 2021. p. 665–74.

[48] Gao C, Wang X, He X, Li Y. Graph neural networks for recommender system. In: Proceedings of the 15th ACM International Conference on Web Search and Data Mining. 2022. p. 1623–5.

[49] Wang X, Ma W, Guo L, Jiang H, Liu F, Xu C. HGNN: hyperedge-based graph neural network for MOOC course recommendation. Inf Process Manag. 2022:59(3);102938.

[50] Ma T, Huang L, Lu Q, Hu S. KR-GCN: knowledge-aware reasoning with graph convolution network for explainable recommendation. ACM Trans Inf Syst 2023;41(1):1–27. doi: 10.1145/3511019

[51] Subramanian S, Rajesh S, Britto PI, Sankaran S. MDHO: mayfly deer hunting optimization algorithm for optimal obstacle avoidance based path planning using mobile robots. Cybern Syst. 2023;1–20.

[52] Tlijani H, Jouila A, Nouri K. Optimized sliding mode control based on cuckoo search algorithm: application for 2DF robot manipulator. Cybern Syst. 2023:1–17.

[53] Wanting L, Yuqing Z. Recommendation model combining RippleNet and KGCN. In: Proceedings of the 9th International Conference on Signal Image Process (ICSIP). 2024. p. 312–8.

[54] Zhang Y, Pang L, Shi L, Wang B. Large scale purchase prediction with historical user actions on B2C online retail platform. arXiv preprint 2014

[55] Qiao P, Zhang Z, Li Z, Zhang Y, Bian K, Li Y, et al. TAG: joint triple-hierarchical attention and GCN for review-based social recommender system. IEEE Trans Knowl Data Eng 2023;35(10):9904–19. doi: 10.1109/tkde.2022.3194952

[56] Vashishth S, Sanyal S, Nitin V, Talukdar P. Composition-based multi-relational graph convolutional networks. arXiv preprint 2019

[57] Zhao J, Wang W, Zhang Z, Sun Q, Huo H, Qu L, et al. TrustTF: a tensor factorization model using user trust and implicit feedback for context-aware recommender systems. Knowl-Based Syst. 2020;209:106434. doi: 10.1016/j.knosys.2020.106434

[58] Nguyen LT, Tran TT. CombiGCN: an effective GCN model for recommender system. In: Proceedings of the International Conference on Computer Data Social Network. 2023:111–9.

[59] Tiukhova E, Penaloza E, Oskarsdottir M, Baesens B, Snoeck M, Bravo C. INFLECT-DGNN: influencer prediction with dynamic graph neural networks. IEEE Access. 2024.

